# Severe *Plasmodium vivax* infection in Korea

**DOI:** 10.1186/s12936-017-1684-4

**Published:** 2017-01-28

**Authors:** Jae Hyoung Im, Hea Yoon Kwon, JiHyeon Baek, Seong Wook Park, Areum Durey, Kyung Hee Lee, Moon-Hyun Chung, Jin-Soo Lee

**Affiliations:** 1 Department of Internal Medicine, Inha University School of Medicine, Incheon, 400-711 South Korea; 2 Department of Emergency Medicine, Incheon, 400-711 South Korea; 3 Department of Radiology, Inha University School of Medicine, 7-206, Shinheung-Dong, Jung-Gu, Incheon, 400-711 South Korea; 4 Department of Internal Medicine, Jeju University Hospital, Jeju, South Korea

**Keywords:** Acute kidney injury, Malaria, Mortality, *Plasmodium vivax*, Pulmonary oedema

## Abstract

**Background:**

Although severe malaria by *Plasmodium vivax* has been increasingly reported, there are marked variations in the type and rate of the complications by geographic area. This is possibly because of the presence of concurrent falciparum malaria or bacteraemia, and of differences in underlying immune status among the infected subjects. Furthermore, published studies on *P. vivax* in temperate regions are limited. The present study investigated severe vivax malaria in Korea, where only vivax malaria occurs. Hence, other compounding factors are rare. Additionally, most of the patients are possibly non-immune to this malarial disease.

**Methods:**

Adults with vivax malaria observed in one 860-bed university hospital from January 2006 to December 2012 were retrospectively evaluated. Seventeen patients who had travelled overseas within 6 months before the presentation of malaria were excluded. Severe vivax malaria was diagnosed according to World Health Organization criteria. Other complications were also investigated.

**Results:**

Two-hundred and ten patients were enrolled, of which 88 (41.9%) were treated as inpatients and the remainder as outpatients. Eleven patients were treated in an intensive care unit; among them, five patients received mechanical ventilation, and one needed extracorporeal membrane oxygenation therapy (ECMO) additionally. Severe vivax malaria was identified in 44 patients (21.0%), and the most common severe complication was pulmonary manifestation (40/188, 21.9%), which was followed by cerebral malaria (5/210, 2.4%), shock (4/210, 1.9%), spontaneous bleeding (3/210, 1.4%), metabolic acidosis (3/210, 3.5%) and acute kidney injury (2/210, 1.0%). Unusual complications, such as splenic infarction (ten patients) and retinal haemorrhage (two patients) were sometimes observed. There were no deaths, but the case involving ECMO was potentially fatal.

**Conclusions:**

*Plasmodium vivax* infection can be severe to be fatal and is frequently associated with various complications in non-immune adults. The frequency of each complication seems to differ from other countries. Hence, further investigation is needed to elucidate the causes and mechanisms responsible for these differences.

**Electronic supplementary material:**

The online version of this article (doi:10.1186/s12936-017-1684-4) contains supplementary material, which is available to authorized users.

## Background

Malaria is a protozoan disease transmitted by *Anopheles* mosquito. The disease presents as an acute febrile illness, characterized by the classic malaria paroxysm, namely, chills and rigours, followed by fever spikes, and then profuse sweating [1]. About 120 types of *Plasmodium* species have been reported, although only five are accepted as human malaria parasites. Notably, *Plasmodium falciparum* and *Plasmodium vivax* cause most of the human infections worldwide [2]. Vivax malaria is typically accompanied by relatively less severe complications than falciparum malaria. However, there have been increasing reports of severe vivax malaria, including respiratory distress [3, 4], acute kidney injury [5, 6] and cerebral malaria [7, 8], which have been attributed to various factors [9]. These reports were mainly from tropical regions, while only a few studies have investigated malaria in temperate regions, such as Europe and Far-east Asia [10–12]. A recent review of severe vivax complications also suggests that geographical heterogeneity may be caused by endemicity and chloroquine resistance [13]. Moreover, additional differences, such as medical facilities, co-morbidity and co-infection, between South Korea and other areas, can contribute to dissimilarities in vivax malaria complications.

In Korea, *P. falciparum* infection was once present among intravenous drug abusers, while indigenous falciparum malaria has not been reported since 1945. *Plasmodium malariae* was also present before 1945, but has not occurred since. In addition, the transboundary movement is impossible in Korea due to the geographic (peninsula) and political situation (North Korea). Thus, misdiagnosis or mixed infection with *P. falciparum* imported from abroad is not possible in South Korean residents with no history of overseas travel. In contrast, vivax malaria had been prevalent in Korea for many centuries, its incidence decreased rapidly from the 1970s. The Republic of Korea (South Korea) was declared to be a malaria-free area in 1979 [14]. However, vivax malaria re-emerged in 1993. It was initially localised to the area around the border with North Korea but has since spread from west to east along the Demilitarized Zone (DMZ), although the endemic area has not yet extended south beyond the neighbouring provinces [15–17]. As indigenous vivax malaria has not occurred for nearly 30 years in most areas of Korea, most Koreans, particularly those under 40 years of age, are vivax malaria-naïve. Thus, the effect of pre-existing specific immunity on the manifestation of malaria can be excluded. Furthermore, the possibility of recurrent or reinfection with *P. vivax* is also very low.

The Korean population has a relatively high socio-economic status and appropriate medical care system. Consequently, other contributing factors, including salmonellosis, dengue fever, and nutritional deficiency, rarely or do not occur, which minimizes the effects of these confounding factors. Additionally, chloroquine resistance to *P. vivax* is rare [17, 18], so the failure of anti-malarial therapy is not an issue. In this context, vivax malaria in Korea is an ideal model to evaluate the clinical manifestations and complications of vivax malaria, unaffected by compounding factors or pre-existing immunity against *P. vivax*. Hence, the present study investigated the type and frequency of severe vivax malaria complications in Korea and the development of risk factors associated with the disease.

## Methods

### Study site and population

**Fig. 1:**
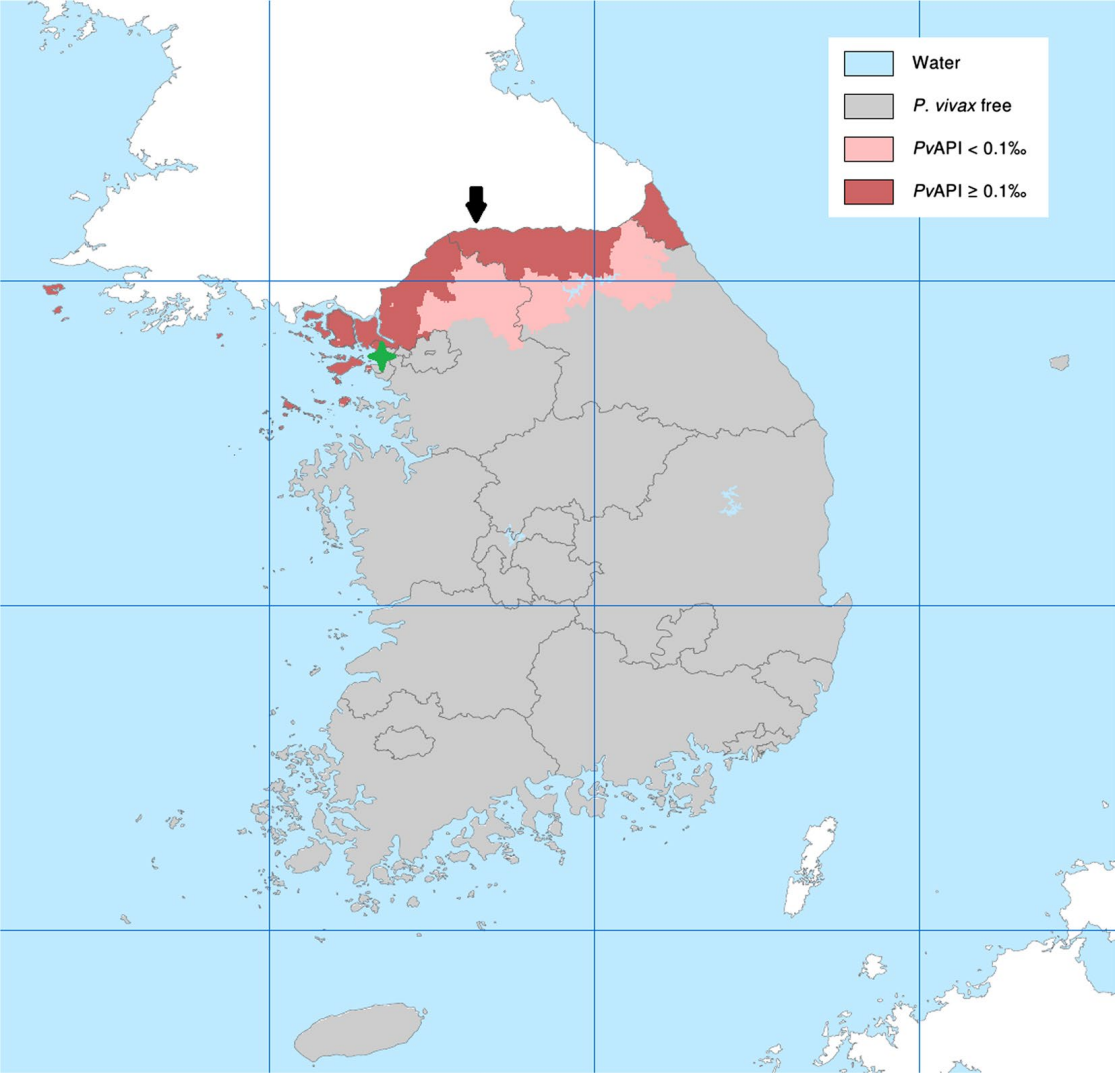
The spatial distribution of *Plasmodium vivax* malaria in Korea and the location of INHA university hospital. *Black arrow* indicates the Demilitarised Zone (DMZ), which is 250 km long and 4 km wide, and serves as a political and military buffer zone between South Korea and North Korea. *Dark red* and *light red* areas indicate the endemicity of vivax in South Korea. The *green star* indicates the location of INHA university hospital

The present study was based at INHA university hospital (Sinheung-Dong, Jung-Gu, Incheon, South Korea) which has 860 beds, a 24-h emergency department and an intensive care unit (ICU) with facilities for mechanical ventilation and extracorporeal membrane oxygenation (ECMO). Incheon has an area of 1010 km^2^ and 2.7 million people with areas endemic for malaria because it is close to the (DMZ) in north-western South Korea. Between 2006 and 2012, The Korea Centre for Disease Control reported 257 and 1406 malaria incidences in Incheon and South Korea, respectively [19]. Figure 1 shows the malaria endemic areas in South Korea [20] and the location of INHA university hospital (37°45′881′′N, 126°63′192′′E).

Both in- and outpatients with vivax malaria, who were diagnosed from January 2006 to December 2012 were included in the study. Their diagnosis was confirmed by peripheral blood smear examination, which was performed by an expert. In order to exclude co-infection with other malarial species imported from abroad, patients who had a history of overseas travel within 6 months before the presentation of the current malaria were excluded. Children were also excluded, for the convenience of comparison with previous reports. The study was conducted by retrospective review of medical records, and demographic and radiologic data. Underlying diseases and laboratory examinations were recorded. Severe malaria was classified according to World Health Organization (WHO) criteria for *P. falciparum* [21], namely consciousness, convulsion (more than 2 per 24 h), hypotension (systolic blood pressure <80 mmHg), bleeding tendency, pulmonary oedema (by chest roentgenogram or computed tomography (CT)), hypoxaemia (oxygen saturation <92% on room air), severe anaemia (haemoglobin <7 g/dL together with a parasite count >10,000/μL), jaundice (serum total bilirubin >3 mg/dL in conjunction with a parasite count >100,000/μL), hypoglycaemia (serum glucose <40 mg/dL), metabolic acidosis (bicarbonate <15 mmol/L or lactate >5 mmol/L), and renal failure (creatinine >3 mg/dL or blood urea >60 mg/dL). Additionally, laboratory data were recorded on muscle enzymes (creatinine phosphokinase), haematuria (by microscopic examination), parasitaemia (by microscopic examination, asexual forms/μL, >10%), alanine aminotransferase, aspartate aminotransferase, uric acid, prothrombin time (PT), and partial thromboplastin time (aPTT). For evaluations of complications other than severe malaria, the presence of bacteraemia [with blood culture using the BACTEC system (BD Diagnostic Systems, Franklin Lakes, NJ, USA)], retinal haemorrhage and other ophthalmologic complications (consult sheets to an ophthalmologist), splenic complications (with CT or ultrasonography) were reviewed.

### Ethics statement

This study was approved by the IRB of INHA University Hospital, Incheon, Korea. All patients’ records were anonymized.

### Statistical analysis

The characteristics of the groups are represented as the mean and standard deviation (SD). For age, parasitaemia and the time taken to visit the hospital from the first onset of symptoms, the median and interquartile range (IQR) was reported because the Shapiro–Wilk test showed that the outcome measures did not have a normal distribution. Furthermore, logistic regression analyses were used to understand the individual risk factors for pulmonary oedema. The following independent variables were measured: age, gender, chronic morbidity, the first onset of symptoms, parasite counts and estimated glomerular filtration rate (eGFR), while there were insufficient events to evaluate shock, spontaneous bleeding, metabolic acidosis and cerebral malaria. Haemoglobin, total bilirubin, platelet counts and glucose were not included in the analyses, as these parameters are not deemed risk factors for pulmonary oedema. For the logistic regression model, platelet and parasite counts were respectively analysed after log transformation. Only independent variables with *P* value <0.2 were considered for the final logistic regression model. The Hosmer–Lemeshow test was used to check the logistic model fitting. Finally, multivariate analysis was performed using an enter method. Data analysis was performed using SPSS statistical software (version 18, SPSS Inc, Chicago, USA).

## Results

### General characteristics of vivax malaria patients

Two-hundred and thirty-one patients were diagnosed with *P. vivax* infection by blood smear. Of these patients, 17 were excluded due to a history of overseas travel within 6 months of presenting with the illness. Four patients under 15 years of age (median age of 11 years) were also excluded. The children were treated in the general ward, none had a severe manifestation of the disease and all were discharged from the hospital uneventfully. Thus, a total of 210 patients with vivax malaria were included in the study. The study included 73.3% (154/210) males and 26.7% (56/210) females. The median age was 39.0 (IQR 23.8–49.0) years. There were 122 outpatients, and 88 (including 11 ICU patients) inpatients. Frequent underlying illnesses were hypertension or diabetes mellitus. There were no pregnant woman or HIV-infected patients (Table 1). The median time taken to visit the hospital from the first onset of symptoms was 7.0 (IQR 4.8–10) days. Two patients had a history of vivax malaria. Most patients were treated with oral chloroquine plus primaquine. If the patients were unable to take oral chloroquine for severe vivax malaria, they received intravenous anti-malarial drug.

**Table 1:** General characteristics of 210 vivax malarial patients

Variables	No. of patients (%)
Age range; years	
15–19	12 (5.7)
20–29	54 (25.7)
30–39	43 (20.5)
40–49	51 (24.3)
50–59	29 (13.8)
>60	21 (10.0)
Gender, male/female	154/56 (73.3/26.7)
Number, hospitalisation/ICU	88/11 (41.9/5.2)
Underlying conditions (N = 203)	
Hypertension	18 (8.9)
Diabetes mellitus	11 (5.4)
Heart failure	2 (1)
Malignancy	1 (0.5)

### Laboratory finding of vivax malaria patients

Leukocytosis (leukocyte count >12,000/μL) was present in 0.5% of the patients and 34.3% showed leukopaenia (leucocyte count <4000/μL), and 29.5% had anaemia (haemoglobin <12 g/dL). The most common laboratory abnormality (91.4% occurrence) was thrombocytopaenia (<150,000/μL), whilst 4.4% (9/204) showed renal failure (creatine >1.5 mg/dL). The levels of transaminases (aspartate transaminase (AST) or alanine transaminase (ALT)) were elevated (> three times normal) in 10.8% of the patients. Coagulopathy (aPTT >60 s or PT international normalized ratio (INR) >1.5) occurred in 2.9% (5/174). Hyperuricaemia (≥10 mg/dL) showed in 1.6% (3/190). The median value of parasitaemia was 3152.5/μL (IQR 1089.8–8285.0). According to microscopic examination of the urine, 2.5% (4/162) showed macrohaemoglobinuria (red blood cell >20 elements per high power field (HPF)) (see Additional file 1).

### Severe complication of vivax malaria (according to WHO criteria)

Among 210 patients, 44 (21.0%) had one or more severe complications of vivax malaria, according to the WHO criteria (Table 4). Pulmonary manifestations presented in 40/183 (21.9%), and mechanical ventilation was used to treat respiratory failure in five patients. There were 5/210 patients (2.4%) with cerebral malaria, while 3/210 (1.4%) with spontaneous bleeding with 4/210 (1.9%) with hypotensive shock. Severe acute kidney injuries occurred at 1.0% (2/204) and metabolic acidosis at 3.5% (3/86). There was no hypoglycaemia, severe anaemia or severe jaundice (Table 2). There were no deaths, but one patient with multi-organ failure was potentially fatal, but successfully recovered after management with ECMO, continuous venovenous filtration (CVVH) and mechanical ventilation, as previously reported [22].

**Table 2:** Complications suggestive of severe vivax malaria

Variables, no. of examinations	No. of patients (%)
Death, 210	0 (0.0)
Cerebral malaria, 210	5 (2.4)
Spontaneous bleeding, 210	3 (1.4)
Shock, 210	4 (1.9)
Pulmonary manifestation, 183	40 (21.9)
Radiologically confirmed, 183	40 (21.9)
Hypoxemia, 127	7 (5.5)
Severe anaemia, 210	0 (0.0)
Jaundice, 207	0 (0.0)
Acute renal failure, 204	2 (1.0)
Hypoglycemia, 206	0 (0.0)
Metabolic acidosis, 86	3 (3.5)
Total severe vivax malaria, 210	44 (21.0)

Shock: systolic blood pressure < 80 mmHg, Hypoxemia: oxygen saturation < 92% on room air, Severe anemia: hemoglobin < 7 g/dL together with a parasite count > 10,000/μL, Jaundice:bilirubin > 3 mg/dL together with a parasite count > 100,000/μL, Metabolic acidosis: bicarbonate < 15 mmol/L or Lactate > 5 mmol/L, Acute renal failure: Cr 3.0 > mg/dL or blood urea > 60 mg/dL, Hypoglycemia: < 40 mg/dL

### Analyses of the hospitalisation and the risk factors for pulmonary oedema

The decision to hospitalise or admit the patients to the ICU was based on the clinical judgement of the attending physicians. Mechanical ventilation was considered when the patient had unresolved hypoxaemia with a supplemental fraction of inspired oxygen. Among 210 vivax malaria patients, 77 were treated in the general wards and 11 in the ICU (including four cases with mechanical ventilation only and one case with ECMO, CVVH and mechanical ventilation). All ICU cases had pulmonary oedema (11 patients) and all patients with shock or cerebral malaria presented pulmonary oedema. Although nine severe malaria patients were treated as outpatients, seven had pulmonary oedema without hypoxaemia. Two separate cases of spontaneous bleeding (gum bleeding) and acute kidney injury were also tolerable (Table 3).

**Table 3:** Complications in outpatients, inpatients, ICU, mechanical ventilator and ECMO cases

Variables	Outpatients, N = 122	Inpatients, N = 88			
		Inpatient, N = 88	ICU, N = 11	Ventilator, N = 5	ECMO, N = 1
Cerebral malaria	0	5	4	2	1
Spontaneous bleeding	1	2	0	0	0
Shock	0	4	4	4	1
Pulmonary manifestation	7	33	11	5	1
Severe anaemia	0	0	0	0	0
Severe jaundice	0	0	0	0	0
Acute kidney injury	1	1	1	1	1
Hypoglycemia	0	0	0	0	0
Metabolic acidosis	0	3	3	2	1
Total severe vivax malaria	9	35	11	5	1

*ICU* Intensive Care Unit, *ECMO* extracorporeal membrane oxygenation, *Inpatients* general ward + ICU, *ICU* included mechanical ventilation and ECMO

In multivariable analyses of the risk factors for pulmonary manifestation, high parasite counts were the greatest risk factor for pulmonary oedema (AOR 2.17 [95% CI 1.15–4.12], *P* = 0.017), and low eGFR was a risk factor for pulmonary manifestation (AOR 0.98 [95% CI 0.95–0.99], *P* = 0.034) (Additional file 2).

### Other complications of vivax malaria

Abdominal CT or ultrasonography was performed on 72 of the patients. Among them, there were 13 cases of hepatomegaly, one case of liver haematoma, 41 cases of splenomegaly, ten cases of spleen infarction (including two cases previously reported [23]) and one case of subcapsular splenic haemorrhage. Two patients presented retinal haemorrhage (including one case previously reported [24]). There was no co-infection with bacteria or fungus in the blood cultures (Table 4).

**Table 4:** Other complications of vivax malarial patients

Variables, no. of examinations	No. of patients
Liver, 72	
Heptomegaly	13
Hematoma	1
Spleen, 72	
Splenomegaly	41
Infarction	10
Subcapsular hemorrhage	1
Retinal hemorrhage	2
Elevated muscle enzyme, 29	6
Other bacteraemia, 81	0
Hyperparasitaemia, 207	0

## Discussion

The present study confirmed that in Korea, adult patients with vivax malaria primarily show the following characteristics; few previous clinical malaria episode; no association with concurrent bacteraemia; extremely rare occurrences of severe anaemia, hypoglycaemia, and acute kidney injury complications; and no or low mortality. However, cerebral and pulmonary manifestations, spontaneous bleeding, shock, and metabolic acidosis are relatively common, as reported previously [11, 12] (see Additional file 3). Similar to the two previous reports in Korea, no deaths occurred in the present study. One study from Europe, investigating imported vivax malaria, also reported very no mortalities [10]. In contrast, the studies in tropical areas, such as India, Indonesia and Pakistan reported 0.3–9.0% mortality [6, 25–35] (see Additional file 4). Although multiple factors could be responsible for the differences in geographic mortality, the most probable cause is the ease of access to medical facilities. Almost all Korean patients can use the public health insurance scheme, which can also decrease the time before admission. For the patient who received ECMO, without such life-supporting equipment, the patient would unlikely to have survived. In addition, rare or lack of other co-infections, which can cause complications associated with vivax malaria, such as typhoid fever, brucellosis and dengue fever, also contribute to the low mortality. These infections are rare or non-existent in Korea. Furthermore, previous studies showed that drug-resistant vivax was associated with severe malaria [31], whereas chloroquine resistance is rarely reported in Korea [18].

The type and relative frequency of severe complications seem to be different to previous reports from tropical or subtropical countries. For instance, severe anaemia was previously reported to be the most common cause of severe malaria in a tropical area [36]. In comparison, no patients also presenting with severe anaemia were found in the present investigation. Falciparum malaria, intestinal helminths and nutritional deficiency are compounding factors in severe anaemia [9], whereas these factors do not exist or occur very rarely present in Korea. Hypoglycaemia or severe jaundice was also absent in the present study. Conversely, pulmonary oedema had a higher incidence in present study than tropical or subtropical areas [6, 25–35]. Frequent radiologic exams are considered an important cause. In the present study, 188 (87%) patients had a radiologic exam, while 15 patients had only pulmonary oedema on chest roentgenogram without hypoxaemia (saturated oxygen <95%). Although this indicates that more pulmonary oedemas could be detected more frequently by routine radiologic examinations, the present study included five cases of mechanical ventilations, which suggests a common occurrence of pulmonary complications is possible and is distinct from that caused by frequent radiologic examinations. In this instance, a low level of immunity, due to no previous exposure to malaria, is considered to be the main cause. Shock and cerebral malaria were also relatively common in the present study, while acute kidney injury presented with low incidence. It is hypothesised that the complications common to the present study might have different pathogeneses compared to the uncommon complications.

Although there are few studies concerning pulmonary oedema of vivax malaria, low eGFR is generally considered to be a risk factor. Accordingly, in the logistic regression model, patients with low eGFR were at risk of pulmonary oedema. However, the present study also revealed that parasite counts were a risk factor for pulmonary oedema. Generally, it has been accepted that parasitaemia is not associated with the severity of vivax malaria [9]. Partial immunity could explain this inconsistency. Patients with low pre-existing immunity to vivax malaria (such as South Korea) could be more sensitive to parasitaemia, resulting in a severe immune reaction that could be associated with the severity of the disease or pulmonary oedema. In contrast, patients with relatively high immunity could be less sensitive to parasitaemia. Most previous studies were performed in areas with relatively high immunity, hence, parasitaemia could not seem to be associated with the severity of vivax malaria. More research is needed to clarify the link between parasitaemia and the severity of vivax malaria.

There are limited comparable published papers on complications in patients with no or low pre-existing immunity to vivax malaria. Yet, in addition to the medical environment, the extent of malaria endemicity and dissimilarities in the criteria of severe complications, a lack of specific immunity may contribute to the various types of severe complications. In particular, international travellers or patients with neurosyphilis receiving induced malaria might be such cases, although these patients also present certain differences to the present cases. Still, a comparison can be made between patients who have received malariotherapy for neurosyphilis and those in the present study that had not suffered from malaria. Malariotherapy has killed as many as 15% of the patients who have received it, which indicates the severity of induced vivax malaria. However, as above-mentioned there are several differences between patients receiving malariotherapy and those in the present study including the presence of underlying disease (neurosyphilis), poor general health in patients with neurosyphilis and a relatively higher inoculation dosage in malariotherapy [37, 38]. Meanwhile, in a survey of 526 patients with vivax malaria in Europe, there were no deaths and only a few severe clinical complications were reported. A total of 312 patients were admitted, 30 patients had complications and seven patients had clinically significant severe disease [10]. Nonetheless, there were several missing data in the complications and the classification and definition of the complications were not described. And, among 554 patients with imported vivax malaria, 234 had taken prophylactic anti-malarial drugs that may have caused fewer complications. In addition, this study includes many immigrants and refugees, expatriates and foreign visitors. Therefore, it is highly possible that many of them are semi-immune to vivax malaria. For the above-mentioned reasons, none of these studies represents the exact situation of vivax malaria in Korea.

A comparison of the study results from various geographical areas is complicated by the heterogeneity of the patients, such as in- or outpatients, duration of illness and frequencies of co-morbidity or co-infection. The present study attempted to elucidate whether the place of management (namely, outpatient, inpatient and ICU) could discriminate between severe and mild vivax malaria. Accordingly, the numbers of patients with severe complications progressively increased from 7.4% (outpatients) to 39.8% (inpatients) to 100% (ICU patients). Thus, the ratios of inpatients/total patients and ICU patients/inpatients might estimate the overall severity of the study patients, which, in the present study, were 41.9 and 12.5%, respectively, and those in Peru were 1.6 and 26.4%, respectively. It can be cautiously speculated, therefore, that physicians in Peru generally managed mild vivax malaria patients (or the actual severity may be milder in Peru), whereas vivax malaria in hospitalised patients in Peru is more severe (possibly because of more restricted hospitalisation). This assumption is compatible with the present result that the overall severity of vivax malaria in Korea is more severe than that in Peru but mortality is lower because of early hospitalisation and subsequently, proper medical management.

Various non-severe complications are reported in vivax malaria, such as splenic (rupture, infarction, haemorrhage) and ophthalmic issues, as well as myocarditis and pancreatitis [24, 39–43]. These reports, however, are usually case reports or case series. Thus, it is not possible to elucidate their overall and relative incidences in vivax malaria. The present study revealed a relatively high incidence of splenic infarction, possibly by frequent use of the abdominal CT scan or ultrasonography for evaluation of fever. There were no splenic ruptures, and all splenic infarctions were resolved after conservative management, without splenectomy or other procedures required. Four patients with splenic infarction had concomitant pulmonary oedema, without any statistically significant association between these two conditions. Retinal haemorrhage was also relatively common in the present study. Retinal haemorrhage is associated with visual disturbance and its incidence is not affected by frequent laboratory or radiographic examinations. Its incidence may represent an actual frequency in vivax malaria in non-immune patients. In the present study, retinal haemorrhage patients did not present any other severe complications, although a correlation between retinal haemorrhage and the severity of vivax malaria has been previously reported [44].

The present research has several limitations. First, our study was performed in a single centre. The generalization of our research as a representation of all temperate regions can be dangerous. Second, it was a retrospective study. However, many variables were inspected, including variables that have not yet been studied. Lastly, the present study did not included pregnant women and children, which results in very rare occurrence of malaria in children and expectant mothers.

## Conclusions

Vivax malaria in Korea can be life-threatening. However, there were no fatalities due to adequate medical management. The types of severe complications of *P. vivax* infections reported in this study may be different from previous studies, hence, additional studies are needed to reveal the underlying cause for these differences.

## Additional files

**Additional file 1.** Laboratory findings of 210 vivax malarial patients.

**Additional file 2.** Univarialbe and multivariable analyses of the risk factors for pulmonary manifestation.

**Additional file 3.** Literature review of severe vivax malaria in South Korea.

**Additional file 4.** Literatures review of severe vivax malaria in tropical or subtropical area (only adult).
